# Biodegradation mechanisms of PBDE − 209: enzymatic, microbial, and omics-driven insights

**DOI:** 10.1007/s10532-026-10405-8

**Published:** 2026-11-11

**Authors:** Viola O. Okechukwu, Oluwasola A. Adelusi, Temitope R. Fagbohun, Patience M. Awafong, Patrick B. Njobeh

**Affiliations:** 1 Department of Biotechnology and Food Technology, Faculty of Science, University of Johannesburg, Doornfontein Campus, P.O. Box 17011, Johannesburg, South Africa; 2 Department of Biomedical Sciences, Faculty of Health Sciences, University of Johannesburg, Doornfontein Campus, P.O. Box 17011, Johannesburg, South Africa

**Keywords:** PBDE − 209, Microbial debromination, Enzymatic biodegradation mechanisms, Multi-omics integration, Biotechnological remediation

## Abstract

Decabromodiphenyl ether (PBDE − 209) is the most extensively produced and environmentally persistent congener among polybrominated diphenyl ethers (PBDEs) and remains widely detected in soils, sediments, sludge, dust, and biota despite global regulatory restrictions. Its extreme bromination, strong hydrophobicity, and resistance to physicochemical degradation limit natural attenuation and complicate remediation efforts. Although biological processes are increasingly recognized as key drivers of BDE − 209 transformation, current knowledge remains fragmented across microbial, enzymatic, and molecular scales, hindering predictive understanding and effective remediation strategies. Unlike previous PBDE reviews that examine microbial taxa or degradation reactions in isolation, this review provides a systems-level synthesis that integrates microbial ecology, enzyme catalysis, metabolic bottlenecks, and multi-omics evidence to explain why PBDE − 209 biodegradation frequently remains incomplete in both natural and engineered systems. Reductive debromination, oxidative transformation, and stepwise formation of lower-brominated intermediates are critically examined alongside with their environmental relevance and toxicity implications. Recent advances in metagenomic, transcriptomics, proteomics, and metabolomics are integrated to identify key functional genes, active enzymes, metabolic constraints, and cooperative microbial interactions governing degradation efficiency. The review further evaluates emerging biotechnological strategies, such as bioaugmentation, biostimulation, engineered microbial systems, immobilized enzymes, synthetic consortia, rhizoremediation, and advanced bioreactor configurations, with emphasis on overcoming bioavailability limitations and minimizing accumulation of toxic intermediate. By consolidating mechanistic insights across biological scales, this review establishes a comprehensive systems-level framework to advance predictive understanding and guide the development of effective, field-applicable remediation strategies for BDE − 209 contaminated environment.

## Highlights

- PBDE-209 biodegradation involves reductive debromination and oxidative transformation.
- Strong sorption and limited bioavailability limit microbial and enzymatic transformation.
- Microbial communities and enzyme systems influence pathway progression and intermediate formation.
- Omics approaches help link microbial function with transformation pathways and metabolic bottlenecks.
- Bioremediation methods require evaluation of pathway completeness, transformation products and toxicity.

## Introduction

Polybrominated diphenyl ethers (PBDEs) are a class of additive brominated flame retardants extensively incorporated into plastics, textiles, electronic housings, polyurethane foams, and construction materials to reduce fire ignition and retard flame propagation (Jinhui et al. 2017; Silva et al. 2021). As PBDEs are physically mixed rather than chemically bound to polymer matrices, they are continuously released throughout product lifecycles, facilitating widespread dissemination across atmospheric, terrestrial, and aquatic environments. Consequently, PBDEs have been detected globally in air, water, soils, sediments, indoor dust, and biota. Among the 209 theoretically possible congeners, decabromodiphenyl ether (BDE − 209) is the predominant component of the commercial deca-BDE formulation and historically accounted for the largest fraction of total PBDE production worldwide (Kumari 2024; Sarkar et al. 2023).

The fully brominated molecular structure of BDE − 209 confers exceptional thermal stability, low volatility, and high resistance to combustion, properties that supported its extensive industrial use (Enyoh et al. 2024; Xiong et al. 2019). However, these same physicochemical characteristics also result in extreme environmental persistence, strong sorption to organic matter and particulates, and limited susceptibility to abiotic and biotic degradation (Sarkar et al. 2023). Although BDE − 209 generally exhibits lower bioaccumulation efficiency than lower-brominated congeners due to its high molecular mass and steric hindrance, it is not environmentally inert. Under environmental and biological conditions, BDE − 209 can undergo stepwise debromination, producing lower-brominated PBDE congeners that are more mobile, bioavailable, and toxic (Kierkegaard 2007; Sarkar et al. 2023). These transformation products often exhibit enhanced endocrine-disrupting, neurotoxic, and developmental effects, thereby increasing ecological risk, defined here as the potential for adverse effects on organisms and ecosystem, and human health risks beyond those associated with the parent compound.

Despite regulatory restrictions and phased bans implemented in the European Union, North America, and several Asian countries, BDE − 209 remains environmentally relevant due to continued release from legacy products (Xu et al. 2024; Zhong et al. 2025). Informal electronic-waste recycling, open burning, mechanical abrasion, weathering of consumer goods, and leaching from landfills and contaminated sediments continue to act as secondary emission sources (Xu et al. 2024). These pathways sustain long-term environmental reservoirs of BDE − 209 and contribute to its persistent detection in indoor dust, urban air, aquatic ecosystems, agricultural soils, and food webs, particularly in regions with limited waste-management infrastructure (Ma 2024). Once released into the environment, BDE − 209 shows an exceptionally strong affinity for soils, sediments, suspended particulates, and indoor dust due to its extreme hydrophobicity (log K_o_w ≈ 10) and negligible water solubility (Malgaretti 2016). This behaviour promotes prolonged environmental retention, limited aqueous mobility, and trophic transfer primarily via particulate-associated pathways (Khatiwada et al. 2026). Human exposure to PBDEs occurs mainly through ingestion of contaminated food and indoor dust, while inhalation of particle-bound PBDEs and dermal contact also contribute, particularly in indoor environments and e-waste recycling settings (Cai et al. 2020; Shakil et al. 2026). Dust ingestion is particularly important for higher-brominated congeners such as BDE − 209, which preferentially partition to indoor particulates due to their low volatility and high hydrophobicity (Meng et al. 2019). Although the acute toxicity of BDE − 209 is generally lower than that of less-brominated congeners, its extreme environmental persistence enables chronic exposure over extended timescales (Cao et al. 2013; Zhao et al. 2018).

Importantly, abiotic and biotic transformation processes can generate debrominated intermediates, including nona-, octa-, hepta-, and hexa-brominated congeners (Munschy et al. 2011). The persistence of BDE − 209 is attributed to its dense bromination pattern, steric shielding of carbon–bromine bonds, and limited enzymatic accessibility, which collectively hinder microbial attack and slow natural attenuation (Zeng et al. 2010). Conventional physicochemical remediation approaches, including photolysis, advanced oxidation processes, thermal treatment, and chemical reduction, often require high energy inputs, generate toxic by-products, or exhibit limited feasibility under field conditions (Chang et al. 2021). Bioremediation is a promising alternative that often requires less energy and may be less expensive than conventional physiochemical remediation strategies, although its effectiveness can be limited by the accumulation of toxic by-products and challenges related with field-scale application (Sarkar et al. 2023).

Microbial and enzymatic systems have demonstrated the capacity for reductive debromination, oxidative modification, and partial aromatic-ring transformation under both anaerobic and aerobic conditions (Wu et al. 2018; Yu et al. 2019). However, degradation efficiencies remain highly variable and are often constrained by strong sorption to environmental matrices, slow desorption kinetics, limited bioavailability, and the accumulation of toxic transformation intermediates, all of which continue to impede effective bioremediation in complex environmental systems (Lu et al. 2022; Ma et al. 2020a, b).

Existing reviews on PBDEs have primarily focused on their environmental occurrence, human exposure pathways, toxicological effects, or general degradation mechanisms across multiple congeners. While several studies describe microbial debromination or enzymatic transformation of PBDEs, these processes are often examined separately, without explicitly linking microbial community structure, enzyme-level catalysis, metabolic pathway progression, and systems-level constraints (Cai et al. 2020; Zhao et al. 2018). While microbial debromination and enzymatic transformation of PBDEs have been reported under both anaerobic and aerobic conditions, these processes are frequently investigated independently, without integrating microbial community composition, enzyme-level catalysis, stepwise metabolic pathway progression, and broader system-level constraints such as bioavailability and sorption dynamics (Wu et al. 2018; Yu et al. 2019).

Consequently, mechanistic linkages between microbial structure, functional gene expression, enzymatic activity, and transformation efficiency remain poorly resolved (Alidoosti et al. 2024). Recent advances in omics-based approaches, including metagenomics, transcriptomics, proteomics, and metabolomics, have provided unprecedented insights into functional genes, enzyme expression patterns, and intermediate metabolite formation during PBDE degradation (Alidoosti et al. 2024; Parveen et al. 2022). However, these datasets are often fragmented across disciplinary boundaries and experimental platforms, and they have not yet been comprehensively integrated to explain why BDE − 209 biodegradation frequently stalls at partial debromination rather than proceeding to complete mineralisation or detoxification (Yu et al. 2019).

This review addresses this critical gap by providing an integrative, systems-level synthesis of BDE − 209 biodegradation that explicitly connects microbial ecology, enzymatic mechanisms, metabolic pathways, and multi-omics evidence across biological scales. By linking molecular structure to enzymatic accessibility, microbial functional guilds, pathway bottlenecks, and the accumulation of toxic intermediates, this review moves beyond descriptive accounts of degradation to elucidate the mechanistic reasons underlying incomplete mineralisation. Furthermore, by contextualising these insights within environmental constraints and emerging biotechnological strategies, this work establishes a conceptual framework for advancing predictive understanding and guiding the development of effective, field-applicable remediation strategies for BDE − 209.

### Physicochemical properties of BDE − 209 relevant to biodegradation

The molecular architecture of BDE − 209 is characterized by the complete substitution of hydrogen atoms on the diphenyl ether backbone with bromine, resulting in a fully brominated and sterically hindered structure (Li et al. 2016). This extensive halogenation confers high molecular mass, strong electron-withdrawing effects, and dense bromine shielding of the aromatic rings, collectively reducing the susceptibility of carbon–bromine bonds to chemical and biological attack (Zeng et al. 2010). Consequently, BDE − 209 demonstrates exceptional resistance to both abiotic and biotic degradation under environmentally relevant conditions (Li et al. 2016; Zhao et al. 2018). BDE − 209 exhibits extremely low aqueous solubility and very high hydrophobicity (log K_o_w ≈ 9–10), which drive preferential partitioning into organic-rich matrices rather than the dissolved phase (Malgaretti 2016). In soils, sediments, sludge, and indoor dust, the compound strongly sorbs to organic matter, black carbon, and particulate surfaces, resulting in limited mobility but prolonged environmental retention (Ayoko & Uhde 2003; Habimana & Sauvé, 2025).

**Fig. 1:**
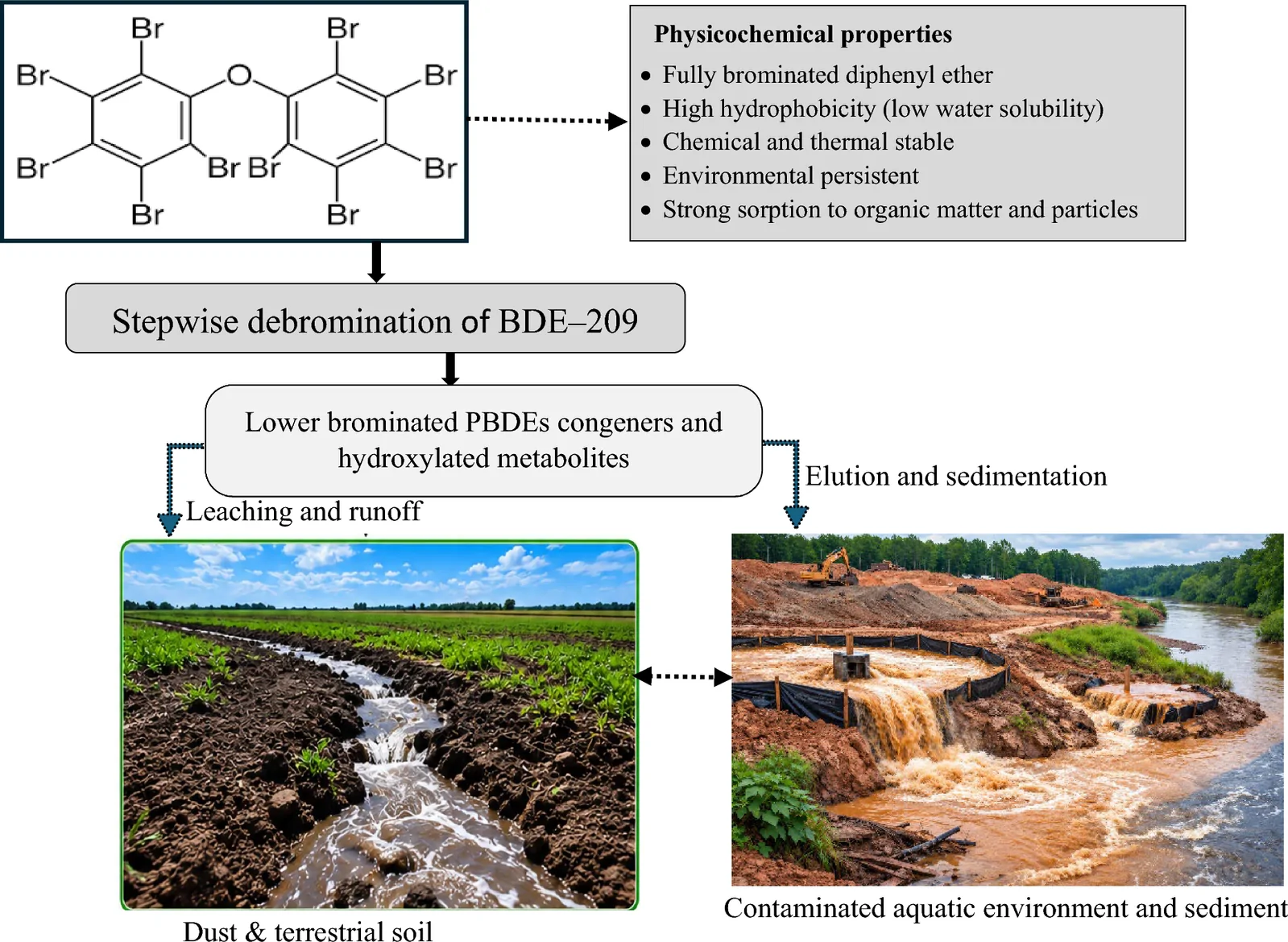
Structural features, transformation pathways and environmental fate of BDE‒209. The solid arrows signify stepwise debromination and the generationof lowerbrominated PBDE congeners and hydroxylated metabolites whereas dotted arrows represent the conceptial transfer and distribution of the transformation products between terrestrial and aquatic environments through processes such as runoff, leaching and sedimentation

This strong sorption behaviour substantially reduces bioavailability, restricting microbial uptake and limiting the accessibility of extracellular enzymes to the contaminant. The physicochemical stability of BDE − 209 further reinforces its persistence. Dense bromination suppresses hydrolytic and oxidative reactivity, while steric shielding limits enzymatic access to reactive sites (Nava & Sarma 2023; Qu et al. 2017). Photolytic debromination may occur under ultraviolet exposure, particularly on exposed particle surfaces; however, sorption to soil and sediment matrices significantly attenuates photolysis in most natural environments (Chang et al. 2020). Redox conditions also influence transformation potential: reducing environments may support slow reductive debromination, whereas oxic conditions generally permit only limited oxidative modification in the absence of specialised enzymatic systems (Fincker & Spormann 2017; Wilsey et al. 2023). Collectively, these physicochemical properties result in long environmental half-lives for BDE − 209, often extending over years to decades in soils and sediments (Fig. 1). Strong sorption, slow desorption kinetics, and limited aqueous availability impose fundamental constraints on biodegradation, such that transformation efficiency is frequently governed more by physicochemical accessibility than by microbial or enzymatic capacity alone (Bher et al. 2022; Kumari & Das 2023). Understanding these constraints is essential for interpreting biodegradation outcomes and for designing remediation strategies that enhance bioavailability and promote effective biological transformation.

### Environmental occurrence and transformation pathways of BDE − 209

BDE − 209 enters the environment primarily through the use, disposal, and degradation of consumer and industrial products containing decabrominated flame retardants (Xu et al. 2024). Major emission sources include leaching and abrasion from plastics, electronic equipment, textiles, and polyurethane foams, as well as informal electronic-waste recycling activities involving manual dismantling, open burning, and uncontrolled disposal (Abogunrin-Olafisoye & Adeyi 2025; Ma et al. 2021; Palaniandy et al. 2022). Landfills and dump sites serve as long-term reservoirs and secondary emission sources, while thermal processes promote atmospheric dispersal via particle-bound emissions, sustaining ongoing environmental loading despite regulatory restrictions (Mathew et al. 2025).

Environmental monitoring studies consistently report BDE − 209 as the dominant PBDE congener in soils, sediments, sewage sludge, indoor dust, and atmospheric particulates (Wu et al. 2019). Elevated concentrations are frequently observed near e-waste recycling sites, urban and industrial areas, and indoor environments containing older electronic or polymeric materials (Ling et al. 2022). In aquatic systems, BDE − 209 rapidly partitions from the water column to suspended particles and sediments, resulting in long-term accumulation in benthic compartments. Atmospheric occurrence is largely confined to particulate-associated forms, enabling regional and long-range transport (Xiao et al. 2025). These patterns reflect the compound’s strong affinity for solid phases and its limited environmental dissipation (Schlesinger et al. 2006; Xiao et al. 2025). Once released, BDE − 209 undergoes slow transformation through a combination of abiotic and biotic processes. Abiotic transformation is dominated by photolytic debromination under ultraviolet irradiation, which initiates stepwise removal of bromine atoms and the formation of lower-brominated congeners (Ma et al. 2020a, b). Photolysis is most effective on exposed surfaces and airborne particulates, whereas sorption to soils, sediments, and organic matrices substantially suppresses photochemical reactivity (Huang et al. 2022). Hydrolysis and chemical oxidation proceed at negligible rates under environmentally relevant conditions.

Biotic transformation represents an environmentally relevant pathway because microorganisms can actively transform BDE − 209 under natural conditions, whereas abiotic processes may be limited by its chemical stability and strong sorption to environmental matrices. Under anaerobic conditions, microbial communities in sediments, sludge, and oxygen-limited soils mediate reductive debromination, generating nona- to hexa-brominated PBDE congeners (Fang et al. 2025; Gerecke et al. 2005; Huang et al. 2014). In contrast, aerobic environments may support oxidative modification, hydroxylation, and limited aromatic-ring destabilisation through bacterial and fungal processes (Reineke & Schlömann 2023; Wu et al. 2018). These transformations increase the polarity and mobility of the parent compound but rarely result in complete mineralization (Du et al. 2021). Instead, lower-brominated and oxygenated intermediates may accumulate in environmental matrices.

Importantly, many transformation products exhibit greater bioavailability and, in some cases, higher toxicological potency than BDE − 209, including enhanced endocrine-disrupting and neurotoxic effects (Dutta et al. 2023; Sarkar et al. 2023). Therefore, environmental transformation does not necessarily equate to detoxification, and partial degradation may redistribute rather than eliminate risk.

## Microbial diversity involved in PBDE − 209 biodegradation: functional guilds and mechanistic roles

Microbial degradation of BDE − 209 is mediated by diverse bacterial and fungal taxa whose functional roles are determined by redox conditions, enzyme repertoires, and ecological interactions (Yu et al. 2019; Zhu et al. 2019). Rather than acting as isolated degraders, microorganisms involved in BDE − 209 transformation function as complementary functional guilds, each contributing discrete but interdependent steps within multi-stage biodegradation pathways. Conceptualising microbial diversity in terms of functional guilds rather than individual taxa provides a mechanistic framework for understanding how degradation proceeds and, crucially, why it frequently stalls at partial transformation in environmental systems (Yu et al. 2019; Zhao et al. 2018). The functional roles and performance of representative microbial, enzymatic, and consortium-based systems involved in PBDE − 209 biodegradation under different environmental conditions are summarised in Table 1.

**Table 1:** Bacterial and fungal oxidative guilds and their extracellular enzyme-mediated transformation pathways

Microbial functional guild	Ecological niche	Extracellular enzymes	Representative fungi	Main substrates	Dominant oxidative mechanisms	Transformation outcomes	References
White-rot fungi	Decaying hardwood and softwood	Laccase, lignin peroxidase, manganese peroxidase versatile peroxidase	*Phanerochaete*, *Trametes*, *Pleurotus*,* Phlebia*	Lignin, PAHs, dyes, PBDEs	One-electron oxidation, radical cascades, aromatic ring cleavage	Extensive depolymerization and mineralization	(Agrawal et al. 2017; Saparrat et al. 2014)
Anaerobic bacterial communities associated with reductive debromination	Anaerobic mangrove sediments	Reductive debromination activity; specific responsible enzymes were not established for individual community members	Mixed bacterial communities from mangrove sediments	BDE − 209	Sequential reductive debromination	Formation of lower-brominated PBDE congeners, including nona- to di-brominated congeners; the study did not establish complete mineralisation	(Yang et al. 2017)
Brown-rot fungi	Coniferous wood, forest soils	Fe^3+^ reducing enzymes, glyoxal oxidase, limited laccase	*Gloeophyllum*, *Postia*, *Serpula*	Cellulose-associated lignin, phenolics	Fenton chemistry (·OH), non-enzymatic oxidation	Partial lignin modification, rapid carbohydrate access	(An et al. 2015; Wei et al. 2010)
Aerobic bacterial degraders	Contaminated soil associated with an e-waste recycling area; laboratory culture	Candidate degradation-associated genes encoding a hydrolase, a dioxygenase and two dehalogenases	*Stenotrophomonas* sp. strain WZN-1	BDE − 209	Debromination; proposed hydroxylation and ring-opening pathways	55.15% BDE − 209 degradation from an initial concentration of 65 μg/L after 30 days under the reported experimental conditions; lower-brominated and oxygenated products were detected or proposed	(Wu et al. 2018)
Soft-rot fungi	Waterlogged wood, soils	Laccase, esterases, cellulases	*Chaetomium, Aspergillus*	Cellulose, hemicellulose	Surface oxidation, ester bond cleavage	Structural weakening of plant tissues	(Díaz-Godínez 2024)
Litter-decomposing fungi	Forest floor, leaf litter	Laccase, manganese peroxidase, oxidases	*Mycena*, *Marasmius*	Leaf lignin, humic precursors	Phenoxy-radical formation, oxidative coupling	Humification, fulvic acid formation	(Zavarzina et al. 2018)
Mycorrhizal fungi	Rhizosphere, plant roots	Laccase, tyrosinase	*Laccaria*, *Pisolithus*	Soil organic matter, phenolics	Selective oxidation, polymer cross-linking	Organic matter stabilization, slow turnover	(Zavarzina et al. 2018)
Endophytic fungi	Plant tissues (leaves, stems, roots)	Laccase, peroxidases, oxidoreductases	Penicillium, Fusarium	Xenobiotics, plant metabolites	Co-metabolic oxidation, detoxification	Reduced toxicity, formation of more polar metabolites	(Díaz-Godínez 2024)
Aerobic bacterial consortium	Laboratory aqueous culture	Intracellular enzyme systems; cytochrome P450 was implicated by inhibitor testing	*Brevibacillus* sp. M1 and *Achromobacter* sp. M2	BDE − 209	Debromination, hydroxylation, ether-bond cleavage and proposed ring-opening processes	88.4% degradation from an initial concentration of 10 mg/L after 120 h under optimised conditions; complete mineralisation and detoxification were not demonstrated by the reported degradation percentage alone	(Hu et al. 2022)
Ectomycorrhizal–saprotrophic intermediates	Forest soils, organic horizons	Laccase, peroxidases	Cortinarius, Hebeloma	Complex soil organic matter	Controlled oxidation, enzymatic priming	Slow SOM turnover, nutrient mobilization	(Zavarzina et al. 2018)
Aquatic fungi (hyphomycetes)	Streams, freshwater litter	Laccase, peroxidases	*Tetracladium, Lemonniera*	Leaf litter lignin, phenolics	Oxidative depolymerization in water	Accelerated litter decay	(Díaz-Godínez 2024)
Extremotolerant/black fungi	Polluted soils, rocks, built environments	Laccase-like oxidases, peroxidases	*Exophiala, Cladophialophora*	Aromatic hydrocarbons, xenobiotics	Slow oxidative transformation	Persistence with partial detoxification	(Díaz-Godínez 2024)

### Primary debrominators: anaerobic reductive specialists

The initial and often rate-limiting step in BDE − 209 biodegradation is reductive debromination, predominantly mediated by strictly anaerobic bacterial guilds. Members of the genus *Dehalococcoides* are among the best-characterised primary debrominators, possessing specialised reductive dehalogenases that catalyse stepwise bromine removal from the fully substituted diphenyl ether backbone under anoxic conditions (Fang et al. 2025). These organisms typically rely on electron donors such as hydrogen or organic acids and couple debromination to energy conservation via dehalorespiration (Lee & He 2010; Zhao et al. 2018). Primary debrominators initiate transformation by converting BDE − 209 into nona- and octa-brominated congeners; however, their metabolic scope is generally restricted to reductive processes. They typically lack the enzymatic machinery required for oxidative modification or aromatic ring cleavage, leading to the accumulation of lower-brominated intermediates when downstream functional guilds are absent or inactive (Zhao et al. 2022). This limitation highlights the dependence of pathway completion on guild succession rather than single-organism activity.

### Facultative and aerobic transformative guilds: oxidative and co-metabolic processors

Following partial debromination, facultative and aerobic bacterial guilds play a critical role in transforming lower-brominated intermediates through oxidative and co-metabolic pathways. Genera such as *Rhodococcus*, *Sphingomonas*, *Pseudomonas*, *Burkholderia*, and *Achromobacter* exhibit metabolic versatility, enabling hydroxylation, partial ring destabilisation, and further modification of PBDE-derived intermediates under oxic or fluctuating redox conditions (Hu et al. 2022; Wu et al. 2018). These organisms generally transform PBDE congeners co-metabolically rather than using them as primary carbon sources, relying on auxiliary substrates to sustain enzyme expression and catalytic activity. Facultative bacteria act as functional bridges between anaerobic and aerobic regimes, adjusting metabolic strategies in response to oxygen availability and redox gradients (Ciemniecki & Newman 2020; Lu & Imlay 2021). Their presence is particularly important in heterogeneous environments such as soils, sediments, and engineered treatment systems, where redox conditions vary spatially and temporally (Yu et al. 2019).

### Fungal oxidative guilds: extracellular enzyme-mediated transformation

Fungal degraders form a distinct aerobic oxidative guild capable of transforming highly brominated aromatic compounds. White-rot fungi such as *Phanerochaete chrysosporium* and *Pleurotus ostreatus* produce extracellular ligninolytic enzymes including laccases, manganese peroxidases, and lignin peroxidases that generate radical species capable of attacking sterically hindered carbon–bromine bonds and destabilising aromatic ring structures (Zhao et al. 2018). Unlike bacterial systems, fungal degradation is not limited by cellular uptake, enabling transformation of particle-bound or poorly bioavailable contaminants. Fungal guilds are therefore particularly relevant in forest soils, composting systems, and engineered bioremediation applications where oxygen and lignocellulosic substrates stimulate enzyme production (Antón-Herrero et al. 2023; Dinakarkumar et al. 2024). However, fungal-mediated oxidation rarely results in complete mineralisation and typically requires integration with bacterial guilds for downstream metabolism of transformation products (Hu et al. 2022).

### Community interactions and consortium-level function

Microbial interactions strongly influence degradation efficiency and pathway completeness in complex environments. Syntrophic relationships are particularly important, with fermentative microorganisms supplying reducing equivalents to dehalogenating bacteria (Pan et al. 2026; Yin et al. 2021), while aerobic and facultative degraders metabolise intermediates released by anaerobic guilds. Differences in substrate affinity, enzyme expression, and environmental tolerance promote niche specialisation and functional partitioning across redox gradients. Mixed microbial consortia derived from e-waste sites, contaminated sediments, and landfill leachates consistently outperform single strains due to their ability to integrate reductive and oxidative processes and mitigate intermediate accumulation (Chakraborty et al. 2025; Yu et al. 2019). These observations show the importance of microbial community succession and functional complementarity in PBDE − 209 biodegradation. (Yu et al. 2019) observed microbial community succession during enrichment of consortium GY1 and documented BDE − 209 debromination. The sequential activities of microbial groups, their interactions, and prevailing environmental conditions may influence the extent of debromination, the transformation of intermediate products, and overall degradation efficiency; however, the specific sequential metabolic roles of individual microbial groups require further investigation.

## Enzymatic degradation mechanisms of PBDE − 209

When fully bioavailable, enzymatic degradation of BDE − 209 is fundamentally constrained by the exceptional strength and steric protection of its carbon–bromine bonds, which are among the most resistant halogen–carbon linkages found in persistent organic pollutants (Cheng et al. 2021). The dense bromination pattern sterically shields the diphenyl ether backbone and withdraws electron density from the aromatic rings, thereby limiting enzymatic accessibility and reducing susceptibility to nucleophilic or oxidative attack (Wischang et al. 2011; Zeng et al. 2010). Despite these constraints, certain microbial enzymes have evolved catalytic strategies capable of initiating debromination and structural modification. These enzymes employ mechanisms such as electron transfer, radical generation, nucleophilic displacement, and oxidative activation to weaken or cleave carbon–bromine bonds, generating the initial intermediates in the overall transformation pathway (Zhao et al. 2018). Enzymatic activation is therefore a critical determinant of degradation potential, as subsequent biotic reactions proceed more readily once bromine atoms are removed or aromatic rings are oxidatively destabilized (Boro et al. 2025).

### Reductive dehalogenation enzymes

Reductive debromination is one of the most important enzymatic routes for transforming the fully brominated structure of BDE − 209 (Zhao et al. 2018). In anaerobic microorganisms, reductive dehalogenases (RDases) catalyse electron-driven cleavage of carbon–bromine bonds using corrinoid cofactors and iron–sulphur clusters to facilitate electron transfer from cellular donors (Zhao et al. 2018). These enzymes typically function within the framework of reductive dehalorespiration, in which debromination is coupled to energy conservation, enabling stepwise conversion of BDE − 209 to nona-, octa-, and lower-brominated congeners (Zhao et al. 2022). The stereochemical arrangement of bromine substituents strongly influences debromination pathways, with para- and meta-substituted positions generally removed more readily than sterically hindered ortho positions (Mancuso & Dalpozzo 2018; Saikia et al. 2016). Reductive pathways therefore dominate in sediments, sludge, and other anaerobic environments where suitable electron donors such as hydrogen or formate are available and typically represent the initiating step in multi-stage biodegradation cascades (Rodríguez‐Salamanca et al. 2022).

Functional differences among PBDE-associated reductive dehalogenases (RDases) highlight the substrate-dependent nature of reductive debromination. In *Dehalococcoides mccartyi* strain GY50, three RDase-encoding genes, *pbrA1*, *pbrA2 and pbrA3*, were highly expressed during PBDE debromination. Comparisons between GY50 and variant strains that had lost a genomic island containing pbrA1 and pbrA2 indicated that these genes were associated with the further debromination of dibrominated products. In particular, the variant strains showed impaired transformation beyond BDE − 15, implicating *PbrA1* and/or *PbrA2* in subsequent debromination steps. *PbrA3* was also implicated in PBDE transformation, although the individual catalytic contributions of these enzymes were not fully established using purified proteins (Ding et al. 2017; Zhao et al. 2021).

Further evidence was obtained from *D. mccartyi* strain TZ50, which contains multiple putative RDase-encoding genes. Native-gel separation, proteomic profiling and in-vitro enzymatic assays implicated PteA({*TZ50*}), TceA({*TZ50*}) and BdeA in PBDE transformation. These enzymes were associated with different stages of the debromination of penta- and tetra-BDE congeners to diphenyl ether, indicating functional diversity among PBDE-associated RDases (Zhao et al. 2018). Nevertheless, the reported enzyme-associated activities principally concern lower-brominated congeners and should not be interpreted as direct evidence that these RDases catalyse the initial debromination of BDE − 209.

Importantly, anaerobic microcosm and sediment studies have independently demonstrated microbial transformation of BDE − 209 into less-brominated congeners, supporting the environmental relevance of reductive debromination (Gerecke et al. 2005; Huang et al. 2014). However, these studies did not identify the specific RDases responsible for the initial transformation of BDE − 209. Identifying and experimentally validating the enzymes responsible for this step therefore remains an important research priority.

### Oxidative and monooxygenase-based enzymes

Complementing reductive mechanisms are oxidative enzymatic processes characteristic of aerobic bacteria or ligninolytic fungi. Laccases catalyze one-electron oxidation of aromatic substrates, generating radical intermediates that destabilise carbon–bromine bonds and facilitate subsequent transformation (Sharma 2025). Peroxidases, including lignin peroxidase and manganese peroxidase, utilise hydrogen peroxide to generate highly reactive oxidised enzyme intermediates capable of attacking hydrophobic aromatic structures, thereby promoting bond cleavage, oxidative coupling, or molecular rearrangement. These oxidative pathways are particularly significant in fungal systems, where the extracellular secretion of ligninolytic enzymes enables the transformation of strongly sorbed or particle-associated PBDEs that exhibit limited cellular uptake (Lu et al. 2021). Such extracellular oxidation represents a critical advantage of fungi in the biotransformation of persistent, highly hydrophobic organic pollutants. In parallel, cytochrome P450 monooxygenases catalyse the introduction of hydroxyl groups into aromatic rings, increasing molecular polarity, reducing hydrophobicity, and enhancing susceptibility to subsequent microbial metabolism (Gordeziani et al. 2016; Guengerich 2018).

### Aromatic ring-cleaving enzymes and advanced transformation

Aromatic ring cleavage represents a later and often rate-limiting stage of PBDE − 209 transformation and typically occurs only after partial debromination or oxidative activation has alleviated the steric and electronic constraints imposed by dense bromination (Zhao et al. 2018). Dioxygenases and related ring-cleaving enzymes can catalyze intradiol or extradiol cleavage of destabilised aromatic rings derived from lower-brominated or oxidised PBDE intermediates, generating linear or partially oxidised metabolites that may enter central metabolic pathways (Wu et al. 2018). The efficiency of aromatic ring-cleavage reactions is determined by enzyme kinetics, substrate affinity, and catalytic turnover, all of which vary substantially among microbial taxa and depend strongly on environmental conditions such as redox status and substrate availability (Hu et al. 2022). Structural and biochemical analyses indicate that hydrophobic substrate-binding pockets, surface-accessible catalytic residues, and cofactor-assisted electron-transfer processes collectively determine the ability of enzymes to accommodate bulky PBDE-derived substrates and catalyse bond cleavage (Zeng et al. 2010). A detailed understanding of these molecular-level interactions is therefore essential for predicting degradation outcomes, guiding enzyme or pathway engineering, and designing bioremediation systems capable of sustaining enzymatic activity and promoting pathway completion for highly brominated pollutants (Zeng et al. 2010; Zhao et al. 2018).

## Metabolic pathways, and toxicological implications of PBDE − 209 transformation

The metabolic transformation of BDE − 209 occurs primarily through stepwise debromination, in which bromine atoms are sequentially removed from the fully substituted diphenyl ether structure (Zhao et al. 2018). This process follows preferential debromination patterns governed by carbon–bromine bond dissociation energies and steric accessibility, with para- and meta-substituted positions generally cleaved more readily than the sterically hindered ortho positions (Zeng et al. 2010). The transformation sequence commonly involves conversion of the parent compound to nona- and octa-brominated congeners, followed by progression to hepta-, hexa-, and penta-brominated derivatives (Yu et al. 2019). These stepwise routes are not strictly linear and may proceed through multiple branching pathways depending on microbial community structure, enzyme specificity, and prevailing redox and substrate conditions (Zhao et al. 2022).

**Fig. 2:**
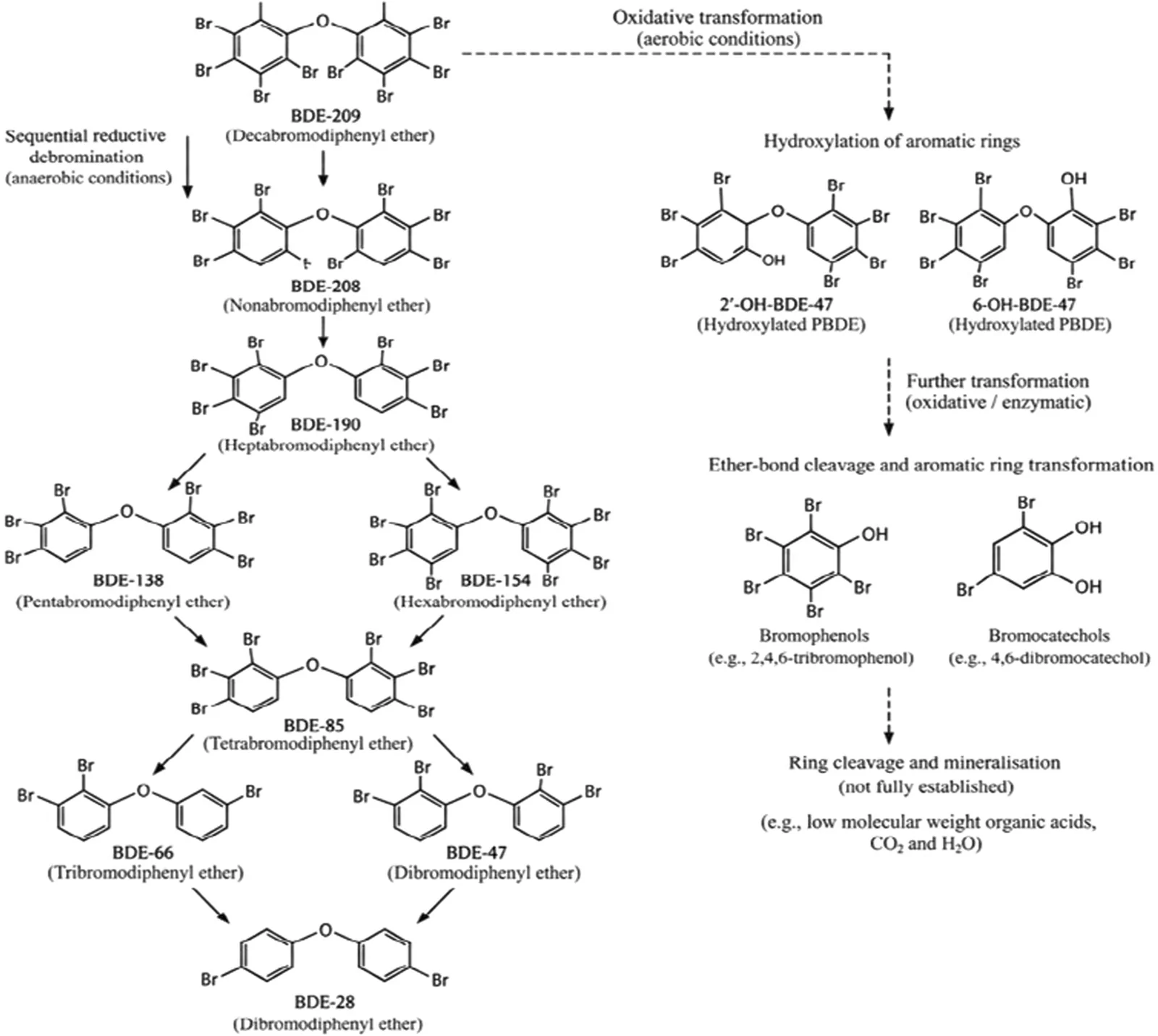
A Schematic diagram illustrating proposed branched degradation pathways for BDE − 209 during microbial degradation

The persistence of intermediate congeners reflects both kinetic limitations and the differential susceptibility of individual carbon–bromine bonds to enzymatic attack (Zhao et al. 2018). Identification of transformation intermediates has revealed a diverse suite of metabolites, including lower-brominated diphenyl ethers, hydroxylated PBDEs, and partially oxidised aromatic compounds (Wu et al. 2018). Advanced analytical techniques, including GC–MS and LC-HRMS, have enabled the detection of these intermediates in laboratory microcosms, contaminated sediments, and engineered bioreactors, providing insight into metabolic progression and pathway completeness (Hu et al. 2022). Lower-brominated congeners generally exhibit increased polarity, reduced molecular mass, and enhanced bioavailability relative to BDE − 209, facilitating subsequent microbial uptake and transformation. In some systems, further oxidation or limited ring-cleavage reactions yield partially mineralised products, including bromophenols, bromocatechols, and low-molecular-weight organic acids (Murnaghan 2023; Wu et al. 2018). Figure 2 illustrates the proposed transformation pathway of BDE − 209, including sequential reductive debromination to lower-brominated PBDE congeners and potential downstream oxidative transformation.

**Fig. 3:**
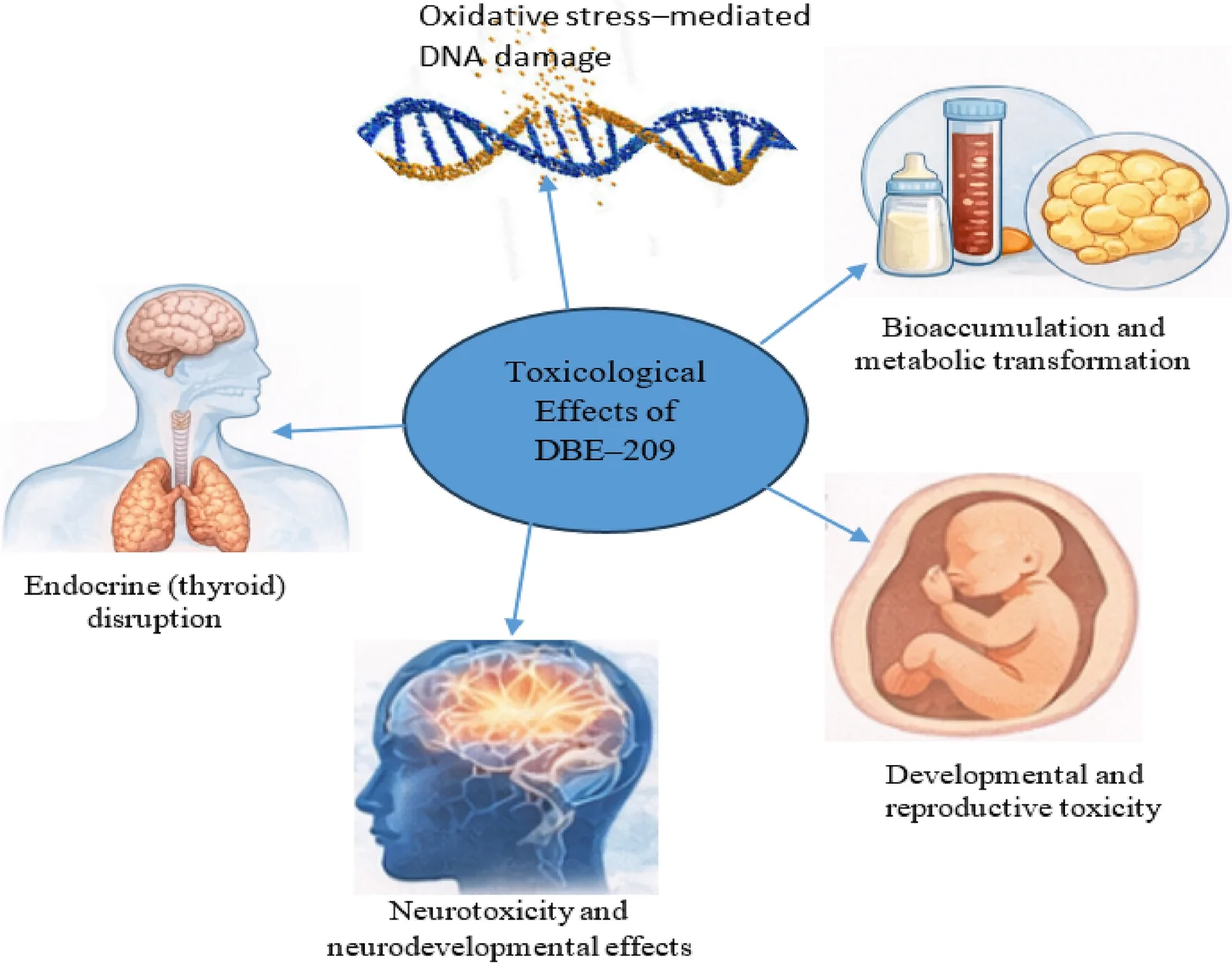
Systematic illustration of toxicological effects of BDE‒209

However, complete mineralisation to carbon dioxide, bromide ions, and water remains rare under environmentally relevant conditions, underscoring the predominance of partial transformation pathways and the accumulation of persistent intermediates (Zhao et al. 2018). The toxicity profile of transformation products therefore represents a critical dimension of metabolic assessment. Lower-brominated congeners and hydroxylated metabolites often exhibit greater toxicity than the parent compound, including improved endocrine disruption, neurotoxicity, and interference with thyroid hormone homeostasis (Fig. 3). Their increased mobility and bioaccumulation potential further elevate ecological and human health risks (Nava & Sarma 2023; Zhao et al. 2018). Therefore, effective bioremediation should aim to promote the further transformation of lower-brominated congeners and other potentially harmful intermediates rather than relying solely on BDE − 209 removal (Yu et al. 2019). Coupling reductive debromination with downstream transformation under suitable environmental conditions may help limit the accumulation of persistent intermediates. Treatment performance should therefore be assessed using both parent-compound removal and the fate and toxicity of transformation products (Yu et al. 2019); (Zhao et al. 2022).

Addressing incomplete BDE − 209 biodegradation requires further studies of how redox conditions, enzyme activity, and microbial community dynamics influence pathway progression and the fate of transformation products. Future studies should determine how the duration and sequencing of anaerobic and aerobic phases affect reductive debromination, subsequent oxidative transformation, and the persistence of lower-brominated congeners. This is particularly important because experimental studies have shown that electron-donor availability influences anaerobic PBDE debromination and microbial community composition, while alternating anaerobic–aerobic treatment has shown potential to improve BDE − 209 degradation under specific experimental conditions (Qiu et al. 2012); (Guo et al. 2026). Research should also identify and experimentally validate the enzymes and functional genes involved in key transformation steps, building on evidence implicating cytochrome P450 activity in aerobic BDE − 209 degradation (Hu et al. 2022). In parallel, microbial community analyses should establish how community succession and interactions relate to transformation efficiency and the persistence of intermediate congeners (Qiu et al. 2012); (Fang et al. 2025). Integrating these findings through multi-omics analyses, chemical identification of transformation products, and toxicity assessment could help guide the design of treatment systems that promote further transformation and reduce the accumulation of potentially harmful intermediates (Guo et al. 2026; Hu et al. 2022). The following sections examine how omics-based approaches can clarify microbial functions and metabolic bottlenecks, and how biotechnological interventions may help address these research needs.

## Omics-based insights into PBDE − 209 biodegradation

High-throughput omics technologies have significantly advanced understanding of the biological processes governing BDE − 209 transformation by resolving functional potential, metabolic activity, and microbial community organization at unprecedented resolution (Xiang et al. 2022). Metagenomic studies have identified gene clusters associated with dehalogenation, oxidation, and other metabolic functions, suggesting that the capacity for BDE − 209 transformation may be distributed across multiple microbial taxa rather than confined to a single organism (Wu et al. 2018; Zhao et al. 2018). However, the presence of these functional genes does not necessarily indicate active biodegradation, because their expression and contribution to transformation depend on environmental conditions, including redox status and substrate availability. Understanding how these factors influence microbial functional potential is therefore important for interpreting biodegradation pathways and predicting transformation outcomes.

Transcriptomic analyses provide dynamic insights into microbial regulatory responses to PBDE exposure, revealing upregulation of dehalogenation, oxidative stress response, and energy metabolism pathways under contaminant pressure (Yu et al. 2019). Expression patterns differ markedly between anaerobic and aerobic systems, with reductive pathways predominating under reducing conditions and oxidative or co-metabolic processes prevailing in oxic environments (Macêdo et al. 2021; Zhao et al. 2018). Proteomic analyses can complement transcriptomic data by identifying proteins associated with BDE − 209 transformation, including candidate enzymes involved in dehalogenation and oxidation. However, protein detection alone does not establish catalytic activity; enzyme activity assays and biochemical validation are needed to confirm their functional roles.

Metabolomic profiling complements genomic and proteomic data by enabling identification of debromination intermediates, hydroxylated congeners, and partially oxidised products that define pathway progression and bottlenecks (Yu et al. 2019). Integrated multi-omics approaches increasingly enable pathway reconstruction, identification of rate-limiting steps, and evaluation of pathway completeness, offering mechanistic insight that surpasses single-technique analyses and supports the development of predictive bioremediation frameworks for highly brominated pollutants (Zhao et al. 2018). Case studies published between 2018 and 2025 demonstrate that combining omics layers yields substantially deeper mechanistic insight than single-technique analyses, particularly in complex microbial consortia (Lu et al. 2025; Makarani & Kaushal 2025). Such integrative frameworks are increasingly essential for translating laboratory observations into predictive models and for guiding the design of effective bioremediation strategies targeting persistent, brominated pollutants.

## Factors influencing biodegradation efficiency of BDE‒209

The environmental distribution, transport, and biodegradation of BDE − 209 are governed by interacting physicochemical and biological factors that collectively determine microbial accessibility and transformation pathways (Yu et al. 2019; Zhu et al. 2019). Environmental parameters such as pH, temperature, and redox potential directly regulate microbial activity, enzyme stability, and reaction kinetics (Gomez et al. 2020; Lowe et al. 1993). Mildly acidic to neutral pH conditions generally favour microbial growth and enzymatic catalysis, whereas strongly acidic or alkaline conditions inhibit dehalogenating and oxidative enzymes or alter transformation routes (Reineke & Schlömann 2023). Temperature controls metabolic rates and enzyme turnover, with moderate ranges promoting effective biodegradation. Redox conditions are particularly critical: anoxic and reducing environments facilitate reductive debromination via electron-transfer reactions, while oxic conditions predominantly support oxidative modification and limited co-metabolic transformation (Wu et al. 2018; Zhao et al. 2018).

**Fig. 4:**
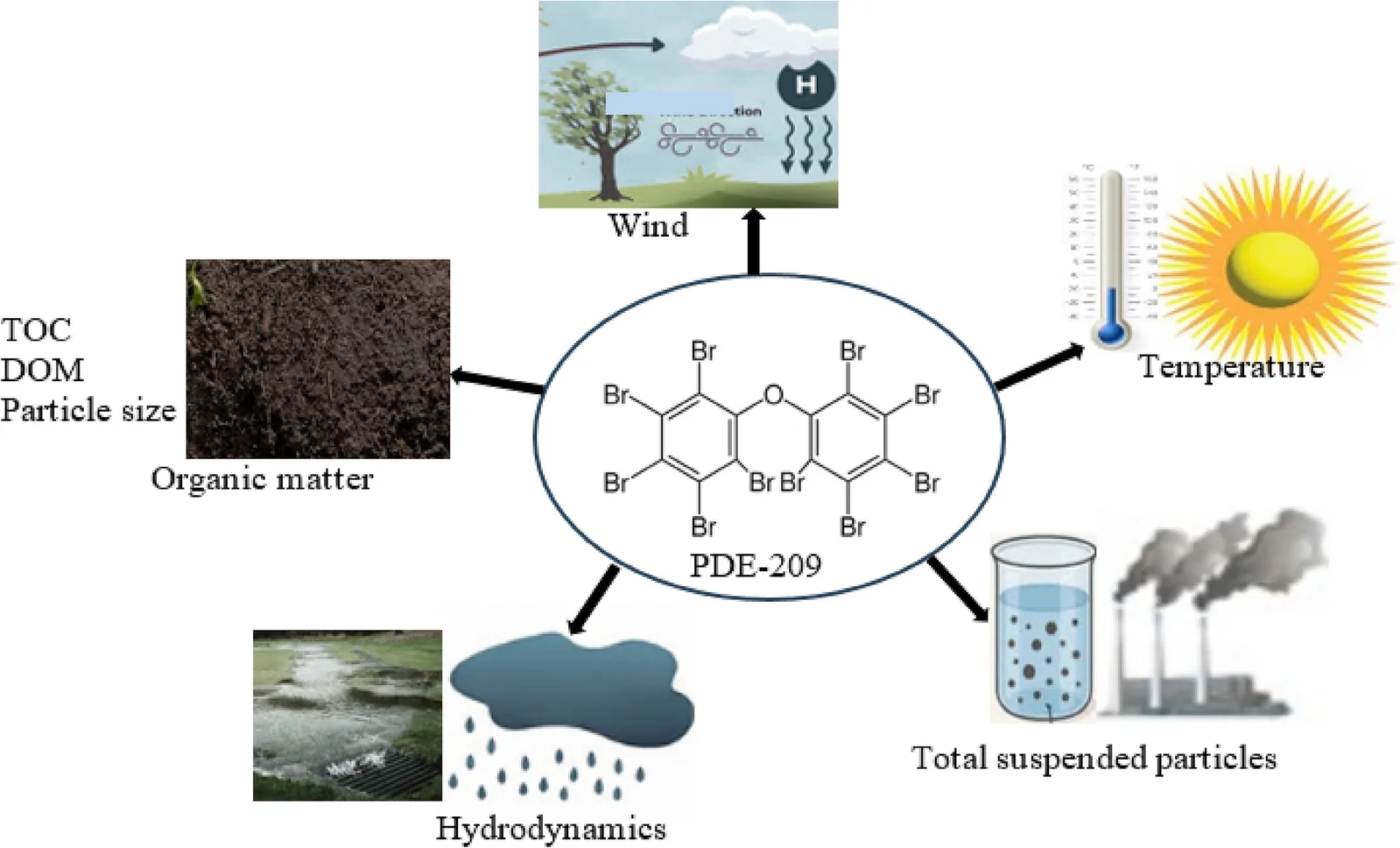
Factors influencing biodegradation efficiency of BD E − 209 (Sun et al. 2024). Total organic carbon (TOC) dissolved organic matter (DOM)

Organic matter exerts a dual influence on biodegradation. Due to its extreme hydrophobicity, BDE − 209 strongly sorbs to organic carbon, suspended particulates, and sediments, substantially reducing dissolved concentrations and limiting microbial uptake (Hale et al. 2020; Zhu et al. 2019). Slow desorption from these matrices frequently represents the primary rate-limiting step in biodegradation (Ni et al. 2014). In contrast, organic matter can indirectly enhance biodegradation by providing carbon sources and electron donors that stimulate microbial metabolism and enzyme production. The net effect depends on organic matter composition, maturity, and spatial distribution (Li et al. 2022). As illustrated in Fig. 4, biodegradation efficiency is influenced by multiple interacting factors, including temperature, organic matter content, hydrodynamics, suspended particles, and nutrient availability.

Electron donors and nutrient availability further modulate biodegradation efficiency by shaping microbial metabolic capacity. Reductive debromination requires sustained reducing equivalents derived from substrates such as hydrogen, organic acids, or fermentable carbon sources, while nitrogen and phosphorus availability influence biomass growth and enzyme synthesis (Lv et al. 2019; Zhao et al. 2018). Co-contaminants can exert both inhibitory and stimulatory effects: toxic metals may suppress microbial activity or deactivate enzymes at elevated concentrations, whereas co-occurring organic pollutants, including polycyclic aromatic hydrocarbons, may stimulate co-metabolic pathways or enrich specialised degraders (Yu et al. 2019).

Ultimately, the environmental matrix integrates these controls. Soils and sediments impose diffusion limitations and heterogeneous redox microenvironments; sludge systems often enhance degradation due to high microbial density and nutrient availability; and aquatic systems promote transport and dilution but limit long-term retention. Strategies that enhance desorption, including the application of surfactants or biosurfactants, have been shown to improve contaminant accessibility and biodegradation efficiency without severe ecological disturbance (Ni et al. 2014; Zhao et al. 2020). A mechanistic understanding of these interacting factors is essential for predicting biodegradation efficiency and for developing sustainable, field-applicable remediation approaches for BDE − 209.

## Biotechnological approaches to enhance BDE − 209 biodegradation

Biotechnological interventions have emerged as essential tools for overcoming the intrinsic recalcitrance of BDE − 209 in both natural and engineered environments (Suarez et al. 2025). Bioaugmentation, which involves the introduction of specialised degrading microorganisms or enriched consortia, has demonstrated potential to accelerate debromination by increasing the abundance of functional degraders at contaminated sites (Qattan 2025). However, its effectiveness is strongly dependent on ecological compatibility, long-term survival, and sustained functional expression within native microbial communities (Yu et al. 2019). In parallel, biostimulation aims to optimise in situ conditions through the addition of electron donors, nutrients, or growth substrates that enhance microbial metabolism and support both reductive and oxidative transformation pathways. Carefully balanced amendments can significantly improve degradation efficiency; however, excessive supplementation may induce microbial competition, oxygen depletion, or secondary environmental impacts (Zhan 2024; Zhao et al. 2018).

Recent advances in microbial engineering offer promising opportunities for targeted enhancement of biodegradation pathways. Engineered strains developed using genome-editing and synthetic biology approaches allow precise manipulation of metabolic networks, enzyme expression, and stress-tolerance traits to improve substrate affinity and catalytic turnover (Adegboye et al. 2021; Fan 2024). Nevertheless, regulatory constraints, ecological risks, and uncertainties associated with environmental release currently limit their field-scale application. As an alternative, immobilised enzyme systems and nanomaterial–enzyme hybrids provide greater operational control by stabilising catalytic proteins, improving contact with hydrophobic substrates, and enabling enzyme reuse (Abdel-Mageed 2025). Although these systems can achieve high conversion efficiencies in controlled settings, they often lack downstream metabolic integration and may result in incomplete detoxification (Liu et al. 2015).

The deliberate design of synthetic microbial consortia represents a systems-based approach to remediation. By integrating complementary anaerobic and aerobic functional guilds, such consortia enable sequential debromination and oxidative modification that individual organisms cannot achieve independently (Hu et al. 2022). Rhizoremediation further extends this concept by exploiting plant–microbe interactions, where root exudates stimulate microbial activity, enhance contaminant mobilisation, and establish redox gradients conducive to transformation (Li et al. 2025). At the process scale, bioreactor systems provide highly controlled environments for optimising biodegradation through regulation of redox conditions, residence times, and substrate delivery, enabling staged treatment that integrates reductive and oxidative processes while minimising the accumulation of toxic intermediates (Chang et al. 2021).

## Conclusion and future perspectives

Understanding the biodegradation of BDE − 209 is essential for mitigating the long-term environmental and health risks associated with its persistence and the formation of toxic transformation products. Recent mechanistic advances have clarified how microbial functional guilds, enzyme-specific pathways, and stepwise debromination sequences collectively govern its environmental fate. The integration of multi-omics approaches has been particularly transformative, enabling direct linkage between genetic potential, enzyme expression, metabolite formation, and pathway completeness, and revealing why biodegradation frequently stalls at partial transformation rather than complete detoxification. These insights have informed the development of emerging biotechnological strategies, including synthetic microbial consortia, immobilised enzyme systems, rhizoremediation, and staged anaerobic–aerobic bioreactors, which offer viable routes for enhanced remediation.

Despite recent progress, several research priorities must be addressed to establish whether complete microbial mineralisation of BDE − 209 is achievable. First, coupled anaerobic–aerobic treatment systems should be investigated to determine whether reductive debromination can be followed by effective oxidation and subsequent metabolism of lower-brominated and oxygenated intermediates. Second, future studies should distinguish parent-compound disappearance from genuine mineralisation by combining congener-specific analysis, metabolite identification, carbon mass-balance assessment and direct measurement of mineralised carbon, using radiolabelled-substrate experiments where feasible. Quantification of released bromide and residual brominated products would provide complementary evidence of debromination and bromine fate. Third, toxicity assessments should establish whether treatment reduces overall biological hazard rather than merely generating or accumulating potentially persistent or toxic intermediates. Integrating these measurements with omics-guided identification of active microbial populations and rate-limiting metabolic steps would help clarify why transformation pathways remain incomplete. Finally, promising systems require validation in environmentally relevant matrices and at appropriate treatment scales before their effectiveness can be established for practical remediation.

## Data Availability

No datasets were generated or analysed during the current study.
